# UICC Staging after Neoadjuvant/Perioperative Chemotherapy Reveals No Significant Survival Differences Compared to Primary Surgery for Locally Advanced Gastric Cancer

**DOI:** 10.3390/cancers14246169

**Published:** 2022-12-14

**Authors:** Rebekka Dimpel, Alexander Novotny, Julia Slotta-Huspenina, Rupert Langer, Helmut Friess, Daniel Reim

**Affiliations:** 1 Department of Surgery, TUM School of Medicine, Technical University Munich, Ismaninger Strasse 22, 81675 Munich, Germany; 2 Institute of Pathology, TUM School of Medicine, Technical University Munich, Ismaninger Strasse 22, 81675 Munich, Germany; 3 Med. Campus III, Institute of Pathology, Johannes Kepler University and Kepler Universitätklinikum, Krankenhausstrasse 9, 4021 Linz, Austria

**Keywords:** gastric/gastroesophageal cancer, perioperative chemotherapy, Lauren histotype

## Abstract

**Simple Summary:**

The aim of this retrospective study is to clarify whether the UICC stages in neoadjuvantly pretreated patients with gastric cancer or a tumor of the gastroesophageal junction can be compared with the UICC stages of patients who underwent primary surgery. We were able to show that they are comparable.

**Abstract:**

Background: The applicability of UICC TNM staging for gastric cancer (GC) patients treated with neoadjuvant chemotherapy (nCTX) and surgery was not yet analyzed in comparison to patients undergoing primary surgery (PS). The purpose of this analysis was to analyze if the prognostic impact of TNM staging after nCTx is comparable with PS. Methods: Data for patients having been treated for GC with or without nCTx between 1990 and 2016 were analyzed. Uni-(URA) and multivariable regression analyses (MRA) were performed to identify predictors. Survival according to the UICC 8th edition stages was analyzed by the Kaplan–Meier method and cox regression analysis. Propensity score matching (PSM) was performed to balance for confounders. Results: 1149 patients with GC were eligible for primary analysis. URA demonstrated age (*p* < 0.0001), tumor localization (*p* < 0.0001), clinical UICC-stage, complications, UICC stage 0, IIB-IIIC, Lauren subtype, grading, and R-stage to be significantly associated with OS. MRA revealed that age, distal tumor localization, more than 25 dissected lymph nodes, UICC stage 0, IIB-IIIC, and Lauren subtype were significantly and independently related to OS. After PSM, survival analyses revealed only a significant difference for pN2/ypN2 (*p* = 0.03), while all other T and N stages were comparable. Conclusion: UICC dependent survival stages do not change significantly after nCTx treatment for GC. Therefore, UICC staging in its present version is applicable to patients undergoing nCTx.

## 1. Introduction

Gastric cancer is one of the most common malignant diseases worldwide, especially in Eastern Asia. Despite declining case numbers in Western countries, a cure remains a therapeutic challenge [1]. Based on computed tomography, endoscopy, endosonography, and diagnostic laparoscopy, treatment decisions regarding neoadjuvant chemotherapy or primary surgery are made. However, Gockel et al. pointed out that staging examinations can lead to over- and under-staging, which may result in insufficient therapies, such as primary surgery for locally advanced gastric cancer. They showed that endosonography has a higher sensitivity and CT a higher specificity for the detection of lymph node involvement. They referred to the CROSS study in which understanding lead to a 13% reduction in 5-year survival [2,3]. One more important question arises whether the clinical TMN classification is prognostically comparable to the pathological TMN classification. A study by Jeong et al. showed that the clinical stage is a good predictor of survival, but is not superior to the pathological TMN stage in prognostic accuracy [4]. It is well known that a high lymph node ratio is associated with poorer survival [5]. In addition, another study showed that a clinically positive lymph node status that can be downgraded to a node negative status by neoadjuvant therapy seems to be prognostically comparable to a primary node negative status [6]. In the West, gastric malignancy is more often diagnosed at an advanced stage compared to Eastern Asia, and it is preferably located in the proximal third of the stomach or the gastro-esophageal junction (GEJ [7]). Therefore, multimodal treatment concepts such as neoadjuvant/perioperative chemotherapy were introduced as a standard of care after demonstrating survival benefits in randomized controlled trials [3,8,9,10,11,12]. Wu et al. have shown that neoadjuvant chemotherapy achieves downstaging of the lymph node status and can confer a survival benefit [13]. Another study was able to show that it is not the clinical stage but the pathological stage that better predicts survival [14]. Unaffected by all these questions, the TNM staging system is the most commonly applied tool to evaluate prognostic outcomes for these patients. The underlying datasets, however, mostly rely on primary resections. It is therefore not clear if TNM staging predicts survival accurately in those patients undergoing multimodal treatment. The latest revision of the TNM/UICC classification system was undertaken in 2017 and all patients having undergone neoadjuvant treatments were omitted in this project, which neglects clinical reality for Western patients. Further, almost all patient cohorts re-evaluated in the UICC 8th edition derived from Eastern Asia. These patients were shown to have different tumor biology, localization, and most importantly undergo upfront surgery followed by adjuvant chemotherapy as an Asian treatment standard when lymph node involvement is proven by histology. These patients were not omitted from analysis in the 2016 restaging project. Therefore, the prognostic statements in regard to the recent TNM staging system (UICC 8th) may not be correct for patients undergoing neoadjuvant chemotherapy in Europe. AJCC tried to introduce a staging proposal for patients having received neoadjuvant therapy. However, this was neither re-evaluated, nor were European data taken into consideration (fact check required). Taking these considerations into account, the present analysis aims to evaluate the prognostic impact of neoadjuvant/perioperative chemotherapy in comparison to those patients having received primary surgery in order to evaluate if classic TNM staging may be applicable for patients undergoing multimodal treatment in a high-volume single center from Germany.

## 2. Materials and Methods

The prospectively documented gastric cancer database at the Surgical Department of TUM School of Medicine, Munich, Germany, was screened for patients having undergone neoadjuvant/perioperative chemotherapy followed by surgery or surgery alone between 1990 and 2016 to identify eligible patients for retrospective analysis. Data obtained from the medical records were transferred to the institutional database as soon as the patients were discharged from inpatient hospital care. Inclusion criteria were: Proximal gastric cancer (Siewert type II (with extension of <3 cm to the distal esophagus), Siewert type III, fundus), location in the body/antrum, curatively intended resections (R0/R1). Exclusion criteria were: Metastatic disease (*n* = 520), R2 resections (*n* = 338), 30-day mortality (*n* = 76), no resection (*n* = 87), multiple data entries (*n* = 151)), and incomplete data (*n* = 1134). All patients underwent multidisciplinary team review ahead of treatment after staging was performed by endoscopy, endoscopic ultrasound, and CT scan. Patients staged cT2 cN + cM0, cT3/cT4 cN_any_ cM0 underwent either neoadjuvant or perioperative chemotherapy (*n* = 624) or primary resection or if the patient refused chemotherapy ahead of surgery (*n* = 525). Patients either received neoadjuvant/perioperative treatment according to one of the following regimens: two preoperative cycles of cisplatin or oxaliplatin/leucovorin/5-FU (PLF/OLF) or three pre- and postoperative cycles of ECX/ECF (MAGIC) or four pre- and postoperative cycles of FLOT. All surgical procedures were performed according to the Japanese gastric cancer treatment guideline including D2-lymphadenectomy. In the case of proximal gastric cancer, the surgical procedure was extended to the distal esophagus until an intraoperatively R0 situation was confirmed by a frozen section. All resected specimens were examined by specialized pathologists, classified according to the TNM-classification, and staged according to UICC recommendations (8th edition). Adjuvant chemotherapy in primarily resected patients was not applied on a routine basis according to the German guideline effective at the respective time, when no postoperative target lesion was detectable. Patients were followed in person for 60 months from the day of surgery every six to twelve months in a dedicated outpatient department (Roman Herzog Comprehensive Cancer Center) by endoscopy and CT scans according to the institutional protocol. Long-term survival (more than 5 years) data were collected based on either additional visits or phone contacts.

In the primary analysis, significant baseline differences and several confounding variables were detected. Therefore, a secondary analysis was staged. This was accomplished by removing all clinical stage I cancers (early gastric cancer) from the primary dataset, as these are usually not eligible for neoadjuvant/perioperative chemotherapy (*n* = 247). Further, potential confounders were balanced by propensity score matching. The clinically most relevant confounders (age, gender, localization) between the groups were matched by a “nearest neighbor” 1:1 matching with a 0.1 caliper. After PSM, which excluded 390 patients, data were re-analyzed accordingly in the secondary analysis.

The following variables were recorded and included in the analysis: gender, age, location (upper, middle, lower third, whole stomach), clinical stages (cT2N0, cT1/cT2cN+, cT3/cT4cN0, cT3/cT4N+), type of chemotherapeutic regimen applied (PLF, OLF, Taxol + PLF, ECF/ECX, FLOT, modified platin-based CTx), type of surgery (esophagectomy, transhiatal gastrectomy, gastrectomy, subtotal gastrectomy), type of required extension (none, luminal/transhiatal, splenectomy, colon, pancreas, others), number of dissected lymph nodes, number of patients achieving D2 lymphadenectomy, postoperative complications (none, Clavien–Dindo Grade I/II and III/IV), pT-(pT0/pT1a/pT1b/pT2/pT3/pT4a/pT4b), pN-(pN0/pN1/pN2/pN3a/pN3b), UICC stages (UICC-0/-IA/-IB/-IIA/-IIB/-IIIA/-IIIB/-IIIC), grading (G1/2, G3/4), R-status (R0/R1), Lauren histotype (intestinal, diffuse, mixed), TRG Becker (1a/1b vs. 2/3—responders and non-responders) [15], and follow-up period with survival status.

Descriptive statistics on demographic and clinical tumor characteristics were calculated as the mean ± standard deviation (continuous variables) and frequencies (categorical variables). Survival time was calculated from the day of surgery to death or last follow up date (at least 60 months after surgery for survivors). The Kaplan–Meier method was applied to estimate survival probabilities stratified by the application of neoadjuvant/perioperative chemotherapy. The log-rank test was performed to compare the estimated survival between the cohorts. Survival prognosticators were analyzed by uni- and multivariable cox regression analyses. Variables entered into the model were age, gender, localization, neoadjuvant/perioperative chemotherapy, UICC-stage, clinical AJCC stage, Lauren histotype, number of dissected lymph nodes, R-stage, grading, and postoperative complications. pT- and pN stages were not included, as these factors are summarized in their respective UICC stages. After univariable analysis, all variables were entered in the multivariable model. Statistical analyses were performed using SPSS version 25 (IBM Inc., Ehningen, Germany). PSM was performed with R and the Match It Plugin (Version 3.1, Vienna, Austria, URL http://www.R-project.org/ (accessed on 1 April 2014)). *p*-values less than 0.05 were considered statistically significant. This retrospective analysis was approved by the local IRB (No. 364/20s; Ethikkommission der Fakultät für Medizin, TUM School of Medicine).

## 3. Results

For this retrospective analysis, the institutional database for gastric cancer patients was screened and identified 3455 patients that were treated by either surgery or chemotherapy, followed by surgery between 1990 and 2016. After removing all cases not fulfilling the defined inclusion criteria (*n* = 2306), 1149 patients were finally analyzed. Overall, 525 patients underwent primary surgery and 624 underwent neoadjuvant/perioperative chemotherapy ahead of surgery. The patient flow diagram is shown in Figure S1.

The analysis of the baseline characteristics showed significant differences between gender distribution, age, tumor localization, surgery type, surgical extension, and Lauren subtypes. There were more advanced cT/pT−, cN/pN+, and UICC stages in the neoadjuvant treatment group. No significant differences were detected regarding postoperative complication rates, grading, and R status. Extensive baseline characteristics are depicted in Table 1.

**Figure 1:**
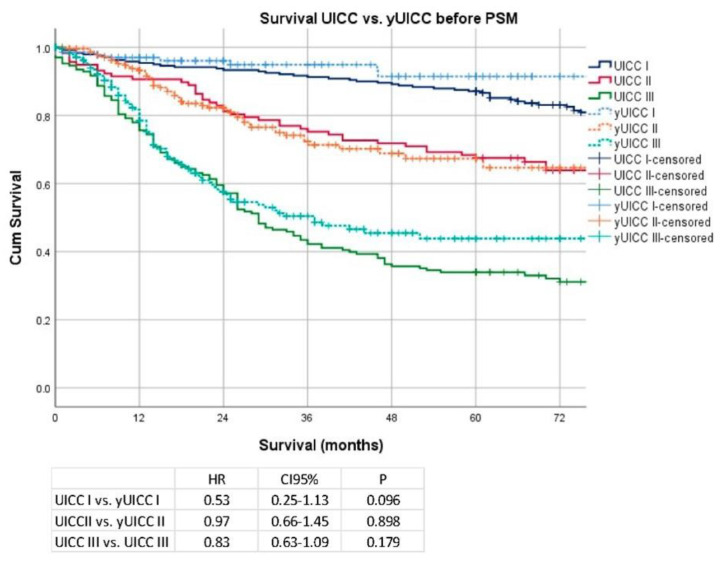
Kaplan–Meier analyses according to UICC/yUICC stages before propensity score matching (PSM).

Median follow-up was 36 months (range 1–199 months), comprising 57 months (range 1–199 months) for survivors and 20 months (range 1–177) months for deceased patients. During the follow-up period, 400 patients (34.8%) died, the five-year survival rate (5YSR) was 62%, and the ten-year survival rate (10YSR) was 40%. Median survival was 114 months (range 1–199) for patients undergoing primary surgery and 120 months (range 1–180) for patients undergoing chemotherapy ahead of surgery (*p* = 0.53) 5YSR/10YSR after primary surgery were 63/49% and 64/57% after neoadjuvant/perioperative chemotherapy followed by surgery. UICC-stage dependent Kaplan–Meier analysis revealed that survival within the respective UICC stages was not different between neoadjuvantly/perioperatively treated or primarily operated patients (UICC I vs. yUICC I: HR 0.53; CI 95% 0.25–1.13; *p* = 0.10; UICC II vs. yUICC II: HR 0.97; CI 95% 0.66–1.45; *p* = 0.90; UICC III vs. yUICC III: HR 0.83; CI 95% 0.63–1.09; *p* = 0.18). Details are shown in Figure 1.

**Figure 2:**
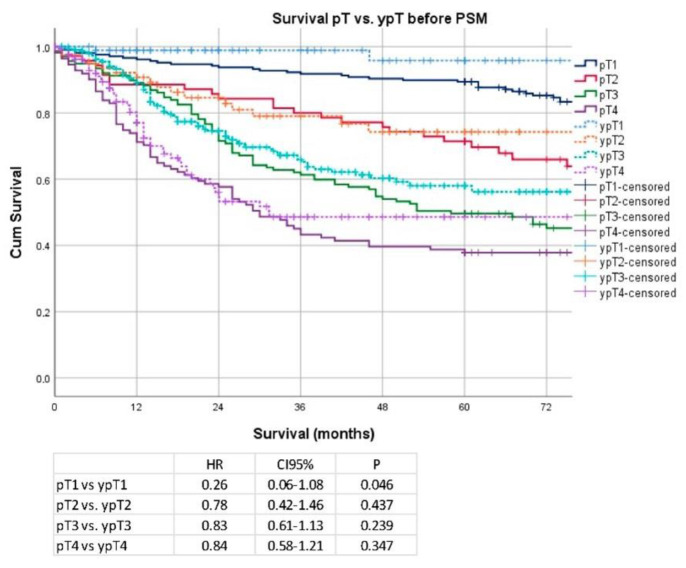
Kaplan–Meier analyses according to pT/ypT stages before propensity score matching (PSM).

**Figure 3:**
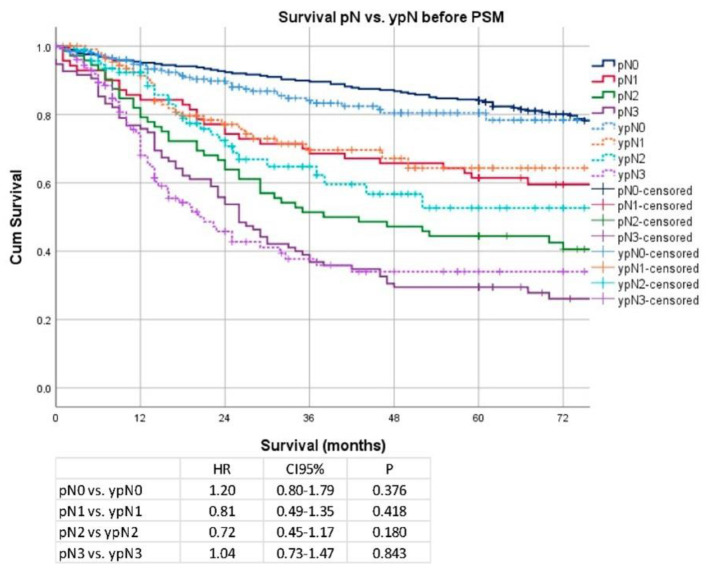
Kaplan–Meier analyses according to pN/ypN stages before PSM.

Analyzing individual pT stages, only stage pT1 vs. ypT1 (median OS 177 months vs. not reached; HR 0.26; CI 95% 0.06–1.08; *p* = 0.05) revealed a weakly significant difference in survival, and stages pT2–pT4 revealed no significant difference in survival (pT2 vs. ypT2: median OS 129 months vs. not reached; HR 0.78; CI 95% 0.42–1.46; *p* = 0.44; pT3 vs. ypT3: median OS 58 vs. 91 months; HR 0.83; CI 95% 0.61–1.13; *p* = 0.24, pT4 vs. ypT4: median OS 30 vs. 31 months; HR 0.84; CI 95% 0.58–1.21; *p* = 0.35). Details are shown in Figure 2. Regarding nodal stages, Kaplan–Meier analyses showed no significant survival advantage in any of the respective stages (pN0 vs. ypN0: median OS 177 months vs. not reached; HR 1.20; CI 95% 0.80–1.79; *p* = 0.38, pN1 vs. ypN1: median OS 92 months vs. not reached; HR 0.81; CI 95% 0.49–1.35; *p* = 0.42, pN2 vs. ypN2: median OS: 38 months vs. not reached; HR 0.72; CI 95% 0.45–1.17; *p* = 0.18, pN3 vs. ypN3: median OS 26 vs. 21 months; HR 1.04; CI 95% 0.73–1.47; *p* = 0.84). Details are shown in Figure 3.

All factors were included in the multivariable model without selection. Univariable regression analysis revealed age, localization, clinical stage, none and severe complications, UICC stage 0, IIB-IIIC, Lauren subtype, grading, and R-stage to be significantly associated to post-therapeutic survival (Table 2. Multivariable analysis demonstrated that age, distal tumor localization, D2-lymphadenectomy, UICC stage 0, IIB-IIIC, and Lauren subtype were significantly and independently related to postoperative survival (shown in Table 3).

### Secondary Analysis after PSM and Removal of Clinical Stage I Patients

The results of the primary analysis revealed significant differences and possible confounders between the respective groups. Further, inclusion of clinical stage I patients may be considered as inappropriate, as these patients would not qualify for neoadjuvant treatment based on pre-therapeutic clinical evaluation. Therefore, all patients demonstrating clinical stage I were removed from the secondary analysis and the most relevant variables for confounding were matched (age, gender, location) by propensity score matching. After removing patients with clinical stage I, the matching algorithm matched 261 patients each (surgery/nCTx + surgery). Analysis of the baseline characteristics demonstrated that the following variables were then well balanced in all groups: gender, age, localization, clinical stage, clinical AJCC stage, number of dissected lymph nodes, Lauren subtypes, complications, and R-stage. The results are shown in Table 4

**Figure 4:**
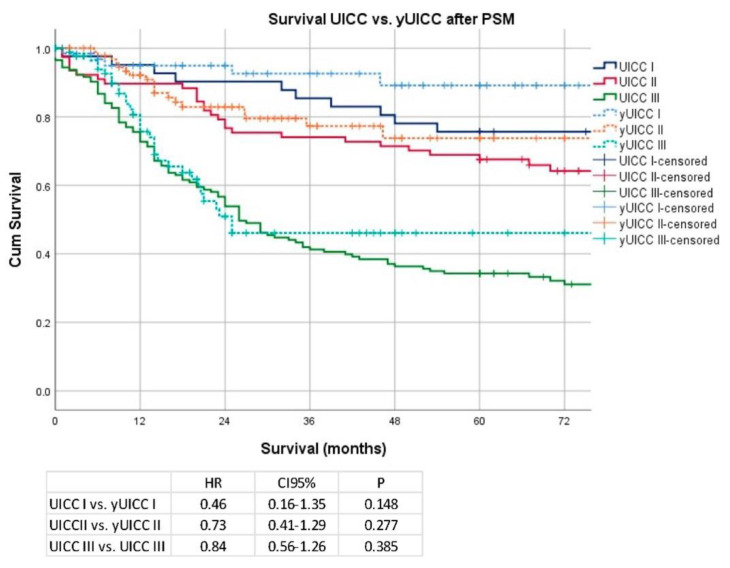
Kaplan–Meier analyses according to UICC/yUICC stages after PSM.

Median follow-up was 29 months (range 1–199 months), comprising 48 months (range 1–199 months) for survivors and 18 months (range 1–149) months for deceased patients. During the follow-up period 214 patients (41%) died, the 5YSR was 56%, and the 10YSR was 39%. Median survival was 67 months for patients undergoing primary surgery, and the median survival time was not reached for patients undergoing chemotherapy ahead of surgery (*p* = 0.01). 5YSR/10YSR after primary surgery and after neoadjuvant/perioperative chemotherapy followed by surgery were 51/34% and 69/59%. UICC stage dependent Kaplan–Meier analysis revealed no significant differences in survival for patients without neoadjuvant and with neoadjuvant treatment (UICC I vs. yUICC I: median OS 149 months vs. not reached; HR 0.46; CI 95% 0.16–1.35 *p* = 0.15, UICC II vs. yUICC II: median OS 93 months vs. not reached; HR 0.73; CI 95% 0.41–1.29; *p* = 0.28, UICC III vs. yUICC III: median OS 26 vs. 25 months; HR 0.84; CI 95% 0.56–1.26; *p* = 0.39). The details are shown in Figure 4.

**Figure 5:**
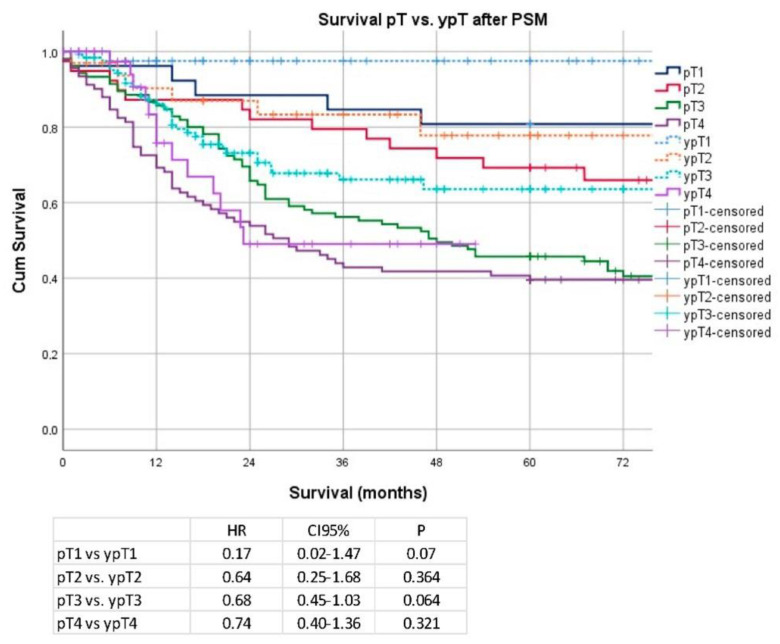
Kaplan–Meier analyses according to pT/ypT stages after PSM.

**Figure 6:**
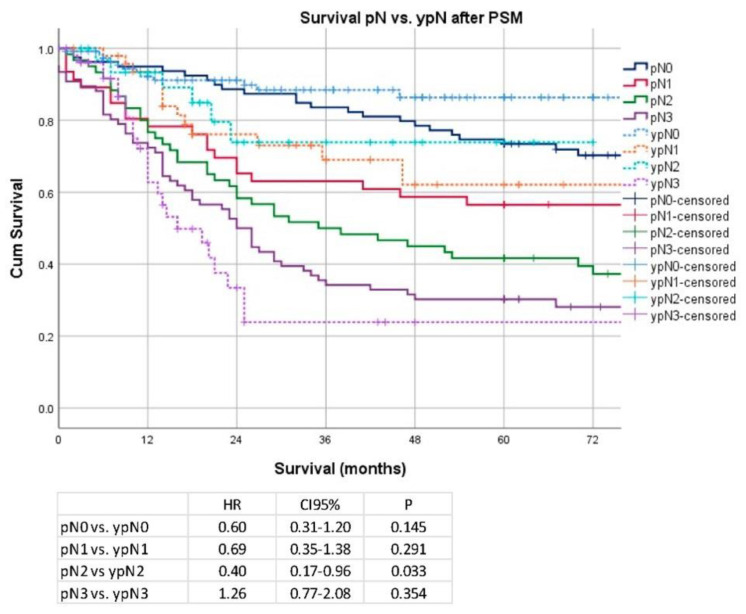
Kaplan–Meier analyses according to pN7ypN stages after PSM.

Regarding the pT/ypT stages, there was no significant difference for stage pT1 vs. ypT1 (median OS 149 months vs. not reached; HR 0.17; CI 95% 0.02–1.47; *p* = 0.07), pT2/ypT2 (median OS 109 months vs. not reached; HR 0.64; CI 95% 0.25–1.68; *p* = 0.36), pT3 and ypT3 (48 vs. 91 months; HR 0.68; CI 95% 0.45–1.03; *p* = 0.06), and pT4/ypT4 (median OS: 29 vs. 23 months; HR 0.74; CI 95% 0.40–1.36; *p* = 0.32). Data are shown in Figure 5. Regarding pN/ypN stages, a significant survival difference was demonstrated only for pN2/ypN2 stage (median OS 38 months vs. not reached; HR 0.40; 0.17–0.96; *p* = 0.03), but no significant differences were detectable for patients without pathological lymph node involvement pN0/ypN0 (median OS 177 months vs. not reached; HR 0.60; CI 95% 0.31–1.20; *p* = 0.15) or other pN stages (pN1/ypN1 (median OS: 92 months vs. not reached, HR 0.69; CI 95% 0.35–1.38; *p* = 0.291) and for pN3/ypN3 stage (median OS 26 vs. 21 months; HR 1.26; CI 95% 0.77–2.08; *p* = 0.35)). Data are shown in Figure 6.

In a further subgroup analysis of the PSM-cohort, histopathologic response rates were analyzed. In UICC stage I, the proportion of responders was highest at 17.8%, in stage II it was 8.8%, and in stage III it was negligible at 3.5%. Data are shown in Table 5.

## 4. Discussion

The aim of the present analysis was to review the applicability of the present UICC classification for gastro-esophageal cancer patients undergoing neoadjuvant chemotherapy. The individual UICC stages were shown to be comparable to the T stages. In the lymph node stages, there was a significant survival difference in the pN2/ypN2 stage, while all other pN/ypN stages were comparable. Histopathologic response was highest in yUICC stage I, which did not translate to a difference in survival prognosis. The present results show that the UICC classification may be applicable and valid for survival prediction for patients undergoing neoadjuvant treatment for gastroesophageal cancer.

The TMN classification and the UICC stages, as defined in the TMN manual, are intended to help the physician to carry out adequate therapy planning, to reflect the prognosis of the patient, to help in the evaluation of tumor treatment, to serve for a standardized exchange of tumor progressions, and for follow-up care. It has been repeatedly adapted and validated over the past decades. As early as 2015, the pathologist Wittekind called for a separate classification for patients undergoing neoadjuvant treatment [16]. Unfortunately, the latest UICC edition did not address this fact. In contrast, the AJCC classification has integrated a separate classification proposal for patients undergoing neoadjuvant treatments in its latest edition. This classification was evaluated in 683 patients from NCDB data between 2004 and 2008. Here, the 5YSR for yUICC I, yUICC II, yUICC III, and yUICC IV are reported to be 76%, 46%, 18.3%, and 5.7%, respectively. In earlier publications by Ajani et al., it was shown that survival was not determined by clinical parameters but by response, pathological tumor stage, and R0 resection [17]. More than this, it was shown that patients who demonstrated initial clinical lymph node involvement and were postoperatively node negative after neoadjuvant treatment were prognostically equivalent to initially negative patients. Furthermore, this study and another showed that patients without lymph node involvement had significantly better survival [6].

Some previous studies already addressed the issue of neoadjuvant therapy for AJCC tumor staging and showed a good prognostic safety [14,18,19,20]. Kim et al. investigated how the addition of the clinical tumor stage contributes to improving prognostic certainty. The data showed that the yp-stage, not the clinical tumor stage, was decisive for prognosis. Furthermore, in 56% of the patients, either an up- or down-staging was detected [14]. A recently published validation study from China, the US, and Italy calls for a further specific breakdown of the stages for a better prognosis and emphasizes the major influence of the lymph node status [20].

The studies on the validation of neoadjuvantly treated patients according to AJCC classification emphasize the need for separate classifications. This distinction is still missing in the UICC classification. There is little literature on the applicability of the UICC classification for patients undergoing neoadjuvant treatments. A previous study on gastro-esophageal tumors has addressed the same issue, showing that the UICC classification was applicable to patients undergoing neoadjuvant treatment and that, as in previous studies, downstaging of lymph nodes provides a decisive survival advantage [21]. Another recent study was able to underline the effect of downstaging by neoadjuvant therapy, showing that patients in stage III without evidence of positive lymph nodes in the histopathological findings have a significantly better survival [13].

The data presented in this analysis demonstrate that the existing UICC classification may be applicable for patients undergoing neoadjuvant chemotherapy for locally advanced gastro-esophageal cancer, as it seems that the final pathological tumor stage determines the prognosis irrespective of the clinical tumor stage. Although randomized trials demonstrated survival benefits for patients undergoing neoadjuvant/perioperative chemotherapies for locally advanced gastric cancer [11,12,22], these beneficial effects did not show better survival in the present Kaplan–Meier analyses. This may be related to the fact that true histopathologic response rates (Becker Ia/Ib) were rather low (3.5–17%). It was shown before that a histopathologic response (HPR) is one of the most important survival prognosticators, and only those patients responding to the neoadjuvant treatment demonstrate survival benefits [23]. Certainly, the new standard of FLOT chemotherapy is underrepresented in the present cohort, which demonstrated that higher HPR rates and novel treatment strategies such as immune checkpoint inhibitor therapy were not analyzed. Additional molecular genomics staging such as TGCA categories may possibly improve survival prediction in the future. However, this was not evaluated in the present analysis and remains speculative.

This analysis has important limitations. These are that this study was monocentric and retrospective. The patient collective is composed of a long observation period and is therefore very heterogeneous with regard to the different surgical and perioperative techniques and the chemotherapy administered. FLOT as a new standard of care is certainly underrepresented in the present data. Critically, PSM may increase the unconscious biases of the two groups and PSM does not balance for unknown factors. Further, mean age was still significantly different after PMS. The matching was related not to mean age but to the age distribution (over/under 70 years), which is why mean age was unaffected after PSM. Nonetheless, it is important to state that patients in the “surgery only” group were older, on average, than those undergoing neoadjuvant chemotherapy, which may have biased/influenced overall survival results. The low number of patients undergoing analysis after PSM does not respect the outcomes of those not being analyzed, and finally, we cannot make any statement about those patients having been omitted by the matching algorithm. The present data may not be adopted for Asian patients due to the known differences of different treatment strategies (early cancers, local/stomach preserving surgery, adjuvant chemotherapy) as much as different biologic and ethnic characteristics. Therefore, this evaluation may be only applicable to western patient populations.

## 5. Conclusions

The present work demonstrates that the existing UICC classification may be applicable for patients undergoing neoadjuvant therapies for gastro-esophageal cancer due to the fact that survival prognosis does not differ significantly between the respective UICC, pT, and pN stages when neoadjuvant treatment was applied. This may be related to the low number of histopathologic responders and the possible centralization effect providing more radical surgery and lymph-node dissection. These results do not imply that patients should no longer receive preoperative chemotherapy but only that the present classification system provides similar prognostic information within the respective tumor stages. These data have to be evaluated prospectively and in a multicentric-/multinational fashion.

## Figures and Tables

**Table 1:** Baselines before PSM.

Before PSM (All)	Surgery Only (*n* = 525)		CTX + Surgery (*n* = 624)		*p*-Value
	*n*	%	*n*	%	
Gender					<0.0001
Female	187	35.62	153	24.52	
Male	338	64.38	471	75.48	
Age	65.3 ± 12.1		59.1 ± 11.1		<0.0001
<70 years	324	61.71	510	81.73	<0.0001
>70 years	201	38.29	114	18.27	
Localization					<0.0001
Proximal	232	44.19	432	69.23	
Middle	127	24.19	98	15.71	
Distal	154	29.33	74	11.86	
Total	12	2.29	20	3.21	
Clinical Staging					<0.0001
cT1	140	26.67	5	0.80	
cT2	159	30.29	54	8.65	
cT3	214	40.76	535	85.74	
cT4	12	2.29	30	4.81	
cN0	288	54.86	104	16.67	<0.0001
cN1	237	45.14	520	83.33	
Clinical Stage AJCC					
I	247	47.05	0	0.00	<0.0001
IIA	52	9.90	59	9.46	
IIB	41	7.81	100	16.03	
III	173	32.95	435	69.71	
IVA	12	2.29	30	4.81	
Clinical Stage					
cT1/cT2 cN−	247	47.05	0	0.00	<0.0001
cT3/cT4 cN0	41	7.81	104	16.67	
cT1/cT2 cN+	52	9.90	59	9.46	
cT3/cT4 cN+	185	35.24	461	73.88	
Type of Surgery					<0.0001
Esophagectomy	15	2.86	134	21.47	
Transhiatal ext. Gastrectomy	156	29.71	293	46.96	
Total gastrectomy	178	33.90	168	26.92	
Subtotal gastrectomy	135	25.71	28	4.49	
Others	41	7.81	1	0.16	
Surgical extension					<0.0001
None	308	58.67	227	36.38	
Luminal/transhiatal	74	14.10	251	40.22	
Splenectomy	17	3.24	17	2.72	
Colon	2	0.38	5	0.80	
Pancreas	3	0.57	16	2.56	
Others	121	23.05	108	17.31	
Dissected LN [Median]	25 [1–76]		29 [5–89]		<0.0001
≤25	248	47.24	209	33.49	0.005
>25	277	52.76	415	66.51	
Complications					0.57
None	405	77.14	465	74.52	
CD I/II	71	13.52	96	15.38	
CD III-V	49	9.33	63	10.10	
pT					<0.0001
pT0	3	0.57	33	5.29	
pT1a	97	18.48	13	2.08	
pT1b	107	20.38	46	7.37	
pT2	70	13.33	83	13.30	
pT3	138	26.29	305	48.88	
pT4a	98	18.67	121	19.39	
pT4b	12	2.29	23	3.69	
pN					0.004
pN0	288	54.86	273	43.75	
pN1	70	13.33	119	19.07	
pN2	72	13.71	99	15.87	
pN3a	66	12.57	95	15.22	
pN3b	29	5.52	38	6.09	
UICC					<0.0001
UICC 0	0	0.00	31	4.97	
UICC IA	185	35.24	45	7.21	
UICC IB	55	10.48	63	10.10	
UICC IIA	61	11.62	119	19.07	
UICC IIB	56	10.67	113	18.11	
UICC IIIA	77	14.67	121	19.39	
UICC IIIB	61	11.62	91	14.58	
UICC IIIC	30	5.71	41	6.57	
UICC 0	0	0.00	31	4.97	<0.0001
UICC I	240	45.71	108	17.31	
UICC II	117	22.29	232	37.18	
UICC III	168	32.00	253	40.54	
Lauren type					<0.0001
Not classified	144	27.43	103	16.51	
Intestinal	268	51.05	296	47.44	
Diffuse	78	14.86	142	22.76	
Mixed	35	6.67	82	13.14	
Grading					0.84
G1/G2	146	27.81	170	27.24	
G3/G4	379	72.19	454	72.76	
R					0.06
R0	482	91.81	552	88.46	
R1	43	8.19	72	11.54	
Histopathologic Response					
Becker Ia/Ib			169	27.08	
Becker II			178	28.53	
Becker III			277	44.39	

**Table 2:** Univariable analysis (all patients).

Univariable (All)	HR	CI 95% Lower	CI 95% Upper	*p*
nCTx	1.04	0.84	1.28	0.720
Gender				0.150
Female	1.00			
Male	1.17	0.94	1.46	
Age				<0.0001
<70 years	1.00			
>70 years	1.85	1.51	2.27	
Localization				
Proximal	1.00			<0.0001
Middle	0.74	0.57	0.96	0.023
Distal	0.59	0.45	0.77	0.000
Total	1.46	0.84	2.55	0.184
Clinical Stage AJCC				
I	1.00			0.000
IIA	1.74	1.18	2.55	0.005
IIB	2.26	1.57	3.26	0.000
III	2.61	1.99	3.42	0.000
IVA	2.56	1.51	4.35	0.001
Dissected LN				
≤25	1.00			0.440
>25	1.08	0.89	1.32	
Complications				
None	1.00			0.013
CD I/II	1.04	0.78	1.39	0.777
CD III-V	1.59	1.17	2.15	0.003
UICC				
UICC 0	1.00			0.000
UICC IA	3.19	0.44	23.13	0.252
UICC IB	4.14	0.56	30.60	0.164
UICC IIA	6.23	0.86	45.13	0.070
UICC IIB	9.85	1.36	71.12	0.023
UICC IIIA	13.44	1.87	96.50	0.010
UICC IIIB	20.73	2.88	148.95	0.003
UICC IIIC	32.61	4.50	236.41	0.001
Lauren type				
Not classified	1.00			0.000
Intestinal	1.59	1.20	2.11	0.001
Diffuse	2.42	1.75	3.33	0.000
Mixed	2.01	1.35	3.00	0.001
Grading				
G1/G2	1.00			0.000
G3/G4	1.58	1.25	2.00	
R				0.000
R0	1.00			
R1	2.85	2.16	3.76	

**Table 3:** Multivariable analysis (all patients).

Multivariable (All)	HR	CI 95% Lower	CI 95% Upper	*p*
nCTx	1.00	0.78	1.29	0.98
Gender				
Female	1.00			
Male	1.02	0.81	1.29	0.85
Age				
<70 years	1.00			
>70 years	1.99	1.60	2.47	0.00
Localization				
Proximal				0.10
Middle	0.77	0.57	1.02	0.07
Distal	0.74	0.55	0.99	0.04
Whole	0.68	0.38	1.23	0.20
Clinical Stage AJCC				
I				0.67
IIA	1.02	0.66	1.58	0.93
IIB	1.10	0.70	1.72	0.69
III	0.91	0.62	1.32	0.61
IVA	0.75	0.41	1.39	0.36
Dissected LN				
≤25	1.00			
>25	0.77	0.62	0.96	0.02
Complications				
None				0.17
CD-I/CD-II	1.04	0.77	1.40	0.79
CD-III/CD-IV	1.36	0.99	1.86	0.06
UICC 0				0.00
UICC IA	2.84	0.38	21.13	0.31
UICC IB	3.89	0.52	28.96	0.19
UICC IIA	5.50	0.76	40.07	0.09
UICC IIB	9.01	1.24	65.36	0.03
UICC IIIA	12.04	1.67	87.10	0.01
UICC IIIB	17.97	2.48	130.12	0.00
UICC IIIC	33.18	4.52	243.67	0.00
Lauren histotype				
Not classified				0.00
Intestinal type	1.75	1.28	2.38	0.00
Diffuse type	1.99	1.41	2.80	0.00
Mixed type	1.67	1.10	2.54	0.02
Grading				
G1/G2	1.00			
G3/G4	1.22	0.92	1.61	0.17
R0	1.00			
R1	1.28	0.94	1.73	0.12

**Table 4:** Baselines after PSM.

After PSM (CS-I Exclude)	Surgery Only (*n* = 261)		CTX + Surgery (*n* = 261)		*p*-Value
	*n*	%	*n*	%	
Gender					0.30
Female	79	30.27	91	34.87	
Male	182	69.73	170	65.13	
Age	66.3 ± 11.9		61.6 ± 11.7		<0.0001
<70 years	156	59.77	174	66.67	0.12
>70 years	105	40.23	87	33.33	
Localization					0.31
Proximal	143	54.79	152	58.24	
Middle	55	21.07	52	19.92	
Distal	56	21.46	44	16.86	
Total	7	2.68	13	4.98	
Clinical Staging					0.67
cT1	6	2.30	4	1.53	
cT2	39	14.94	32	12.26	
cT3	204	78.16	215	82.38	
cT4	12	4.60	10	3.83	
cN0	38	14.56	53	20.31	0.11
cN1	223	85.44	208	79.69	
Clinical Stage AJCC					0.38
IIA	45	17.24	36	13.79	
IIB	38	14.56	51	19.54	
III	166	63.60	164	62.84	
IVA	12	4.60	10	3.83	
Clinical Stage					0.177
cT3/cT4 cN0	38	14.56	53	20.31	
cT1/cT2 cN+	45	17.24	36	13.79	
cT3/cT4 cN+	178	68.20	172	65.90	
Type of Surgery					<0.0001
Esophagectomy	13	4.98	45	17.24	
Transhiatal ext. Gastrectomy	115	44.06	104	39.85	
Total gastrectomy	95	36.40	93	35.63	
Subtotal gastrectomy	30	11.49	18	6.90	
Others	8	3.07	1	0.38	
Surgical extension					<0.0001
None	118	45.21	120	45.98	
Luminal/transhiatal	44	16.86	86	32.95	
Splenectomy	13	4.98	6	2.30	
Colon	2	0.77	4	1.53	
Pancreas	2	0.77	7	2.68	
Others	82	31.42	38	14.56	
Dissected LN [Median]	28 [1–76]		28 [6–70]		0.79
≤25	95	36.40	94	36.02	1.00
>25	166	63.60	167	63.98	
Complications					0.35
None	208	36.40	194	74.33	
CD I/II	29	63.60	37	14.18	
CD III-V	24	9.20	30	11.49	
pT					<0.0001
pT0	1	0.38	14	5.36	
pT1a	5	1.92	9	3.45	
pT1b	20	7.66	19	7.28	
pT2	39	14.94	35	13.41	
pT3	106	40.61	130	49.81	
pT4a	78	29.89	46	17.62	
pT4b	12	4.60	8	3.07	
pN					0.001
pN0	79	30.27	120	45.98	
pN1	46	17.62	52	19.92	
pN2	60	22.99	35	13.41	
pN3a	52	19.92	39	14.94	
pN3b	24	9.20	15	5.75	
UICC					<0.0001
UICC 0	0	0.00	14	5.36	
UICC IA	18	6.90	21	8.05	
UICC IB	23	8.81	28	10.73	
UICC IIA	40	15.33	51	19.54	
UICC IIB	37	14.18	52	19.92	
UICC IIIA	68	26.05	41	15.71	
UICC IIIB	49	18.77	37	14.18	
UICC IIIC	26	9.96	17	6.51	
UICC 0	0	0.00	14	5.36	<0.0001
UICC I	41	15.71	49	18.77	
UICC II	77	29.50	103	39.46	
UICC III	143	54.79	95	36.40	
Lauren type					0.94
Not classified	41	15.71	42	16.09	
Intestinal	125	47.89	118	45.21	
Diffuse	63	24.14	66	25.29	
Mixed	32	12.26	35	13.41	
Grading					0.03
G1/G2	45	17.24	67	25.67	
G3/G4	216	82.76	194	74.33	
R					0.79
R0	227	86.97	230	88.12	
R1	34	13.03	31	11.88	
Histopathologic Response					
Becker Ia/Ib			77	29.50	
Becker II			67	25.67	
Becker III			117	44.83	

**Table 5:** HPR rates (according to Becker) depending on yUICC stage.

	yUICC I		yUICC II		yUICC III		*p* < 0.001
	*n*	%	*n*	%	*n*	%	
Responder	45	17.24	23	8.81	9	3.45	
Non-responder	18	6.90	80	30.65	86	32.95	

## Data Availability

The data presented in this study are available on request from the corresponding author. The data are not publicly available due to European data protection regulation.

## Supplementary Materials

The following supporting information can be downloaded at: https://www.mdpi.com/article/10.3390/cancers14246169/s1, Figure S1: Patient inclusion flow diagram.
