# Supplementary figures and images for: UICC Staging after Neoadjuvant/Perioperative Chemotherapy Reveals No Significant Survival Differences Compared to Primary Surgery for Locally Advanced Gastric Cancer
Source: Cancers (Basel). 2022 Dec 14;14(24):6169. doi: 10.3390/cancers14246169 (PMC9777228; doi:10.3390/cancers14246169)

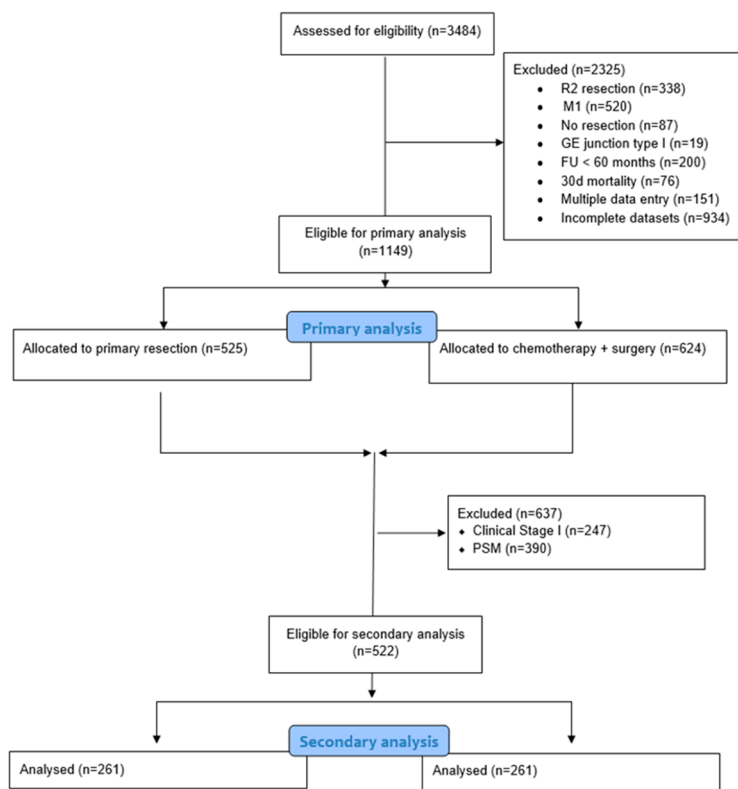

**Figure S1.** Patient inclusion flow diagram.

Supplement: Supplementary file 1 [file cancers-14-06169-s001.zip › cancers-2005737-supplementary.pdf]
